# Impact of prescribed opioid use on development of dementia among patients with chronic non-cancer pain

**DOI:** 10.1038/s41598-024-53728-3

**Published:** 2024-02-09

**Authors:** Tak Kyu Oh, In-Ae Song

**Affiliations:** 1 Department of Anesthesiology and Pain Medicine, Seoul National University Bundang Hospital, Gumi-ro, 173, Beon-gil, Bundang-gu, Seongnam, 13620 South Korea; 2 Department of Anesthesiology and Pain Medicine, College of Medicine, Seoul National University, Seoul, South Korea

**Keywords:** Dementia, Analgesics, Opioid, Pain, Diseases, Risk factors

## Abstract

We aimed to examine the association between opioid use and the development of dementia in patients with chronic non-cancer pain in South Korea. Data were extracted from the National Health Insurance Service database in South Korea. Adult patients diagnosed with musculoskeletal diseases with chronic non-cancer pain between 2010 and 2015 were included in the analysis. Patients who were prescribed opioids regularly and continuously for ≥ 90 days were classified as opioid users. In total, 1,261,682 patients with chronic non-cancer pain were included in the final analysis, of whom 21,800 (1.7%) were opioid users. From January 1, 2016 to December 31, 2020, 35,239 (2.8%) patients with chronic non-cancer pain were newly diagnosed with dementia. In the multivariable model, opioid users showed a 15% higher risk of developing dementia than the control group. Additionally, opioid users showed a 15% and 16% higher risk of developing Alzheimer’s disease and unspecified dementia, respectively, than the control group, but did not show any significant differences for vascular dementia. Among adult patients with chronic non-cancer pain, opioid users were at a higher risk of developing dementia than the control group; the risk was significantly higher for Alzheimer’s disease but not for vascular dementia in this study. Our results suggest that in patients with CNCP, public health strategies should target opioid users for early dementia detection and intervention.

## Introduction

Dementia is a well-known syndrome characterized by the impairment of mental processes, including memory, thinking, reasoning, and judgement^1^. This syndrome significantly affects an individual’s ability to perform activities of daily living^2^. From 1980 to 2009, the prevalence of dementia was 5–7% in most world regions^3^. Dementia management is a critical public health concern, with the estimated average annual cost per person with dementia being €32,506.73 in Europe and €42,898.65 in the United States^4^.

Opioids are the most commonly prescribed analgesics for patients with chronic non-cancer pain (CNCP),^5^ and their prescription rates have continuously increased in many countries, such as the United States,^6^ the United Kingdom,^7^ and South Korea^8^. Opioid use has many adverse effects, one of which is related to the central nervous system (CNS)^9^. Opioids can induce cognitive impairment by modulating several cognitive processes of the mu- and kappa-opioid receptor ligand systems^10^. Moreover, opioid therapy is neurotoxic owing to several cellular and molecular mechanisms^11^, and could induce the development of dementia, such as Alzheimer's disease (AD) or vascular dementia (VD). Opioids are known to disrupt the normal functioning of the hippocampus, which is related to memory and learning^12^, and could alter the levels of neurotransmitters such as serotonin and dopamine, potentially affecting cognitive function^13^. However, findings regarding the relationship between opioid exposure and dementia risks have been inconsistent^14–18^, and this remains a controversial issue.

Therefore, we aimed to examine the association between opioid use and the development of dementia in patients with CNCP in South Korea.

## Methods

### Ethical statement

All procedures were performed according to the ethical standards of the national and institutional committees on human experimentation as well as the principles of the Helsinki Declaration of 1975, revised in 2008. The study protocol was approved by the Institutional Review Board (IRB) of the Seoul National University Bundang Hospital (IRB approval number: X-2105-685-901). The National Health Insurance Service (NHIS) Ethics Committee also agreed to provide data after approval of the study protocol (NHIS approval number: NHIS-2021-1-615). The requirement for informed consent was waived by the IRB of Seoul National University Bundang Hospital as the data were analyzed after masking of the participants and concealment of sensitive information pertaining to the study population.

### NHIS database

The data were obtained from the South Korean NHIS database. As the only public health insurance system in South Korea, the NHIS database includes data on all disease diagnoses and prescriptions for procedures and drugs. Disease diagnosis information was recorded using the International Classification of Diseases, 10th Revision (ICD-10) codes for patients to receive financial support from the government. Additionally, the NHIS database contains demographic and socioeconomic status-related information for all patients in South Korea.

### Patients with CNCP

Patients who were diagnosed with musculoskeletal disorders (MSDs) and visited outpatient clinics or hospitals were considered to have CNCP. The MSDs included rheumatoid arthritis, osteoarthritis, and low back and neck pain. The ICD-10 codes of all MSDs (Supplemental Digital Content 1) were used to extract them from the database and identify all patients with CNCP in South Korea. MSDs were classified into six groups using ICD-10 codes based on the Global Burden of Disease Study 2013^19^.

A medical record technician initially screened all patients registered in the NHIS database between 2010 and 2015 using MSD ICD-10 codes. Data on approximately 500,000,000 patients recorded over a 6-year period were extracted from the database. Owing to the immense sample size, after discussion with other researchers, the technician decided to use a sampling technique. Finally, data on 2.5% of adult patients (aged ≥ 20 years) with CNCP were extracted using a stratified random sampling technique that considered the overall sample size. Age and sex were used as exclusive strata for sampling, and the patients were drawn from each stratum, with sample sizes proportional to the strata of all patients. Through the sampling process, the sex proportion and age distribution of the extracted 2.5% of the sampled patients were similar to those of the overall patient population. A total of 11,593,249 adult patients were sampled, and stratified random sampling was performed using SAS (version 9.4; SAS Institute, Cary, NC, USA)^20^.

**Figure 1:**
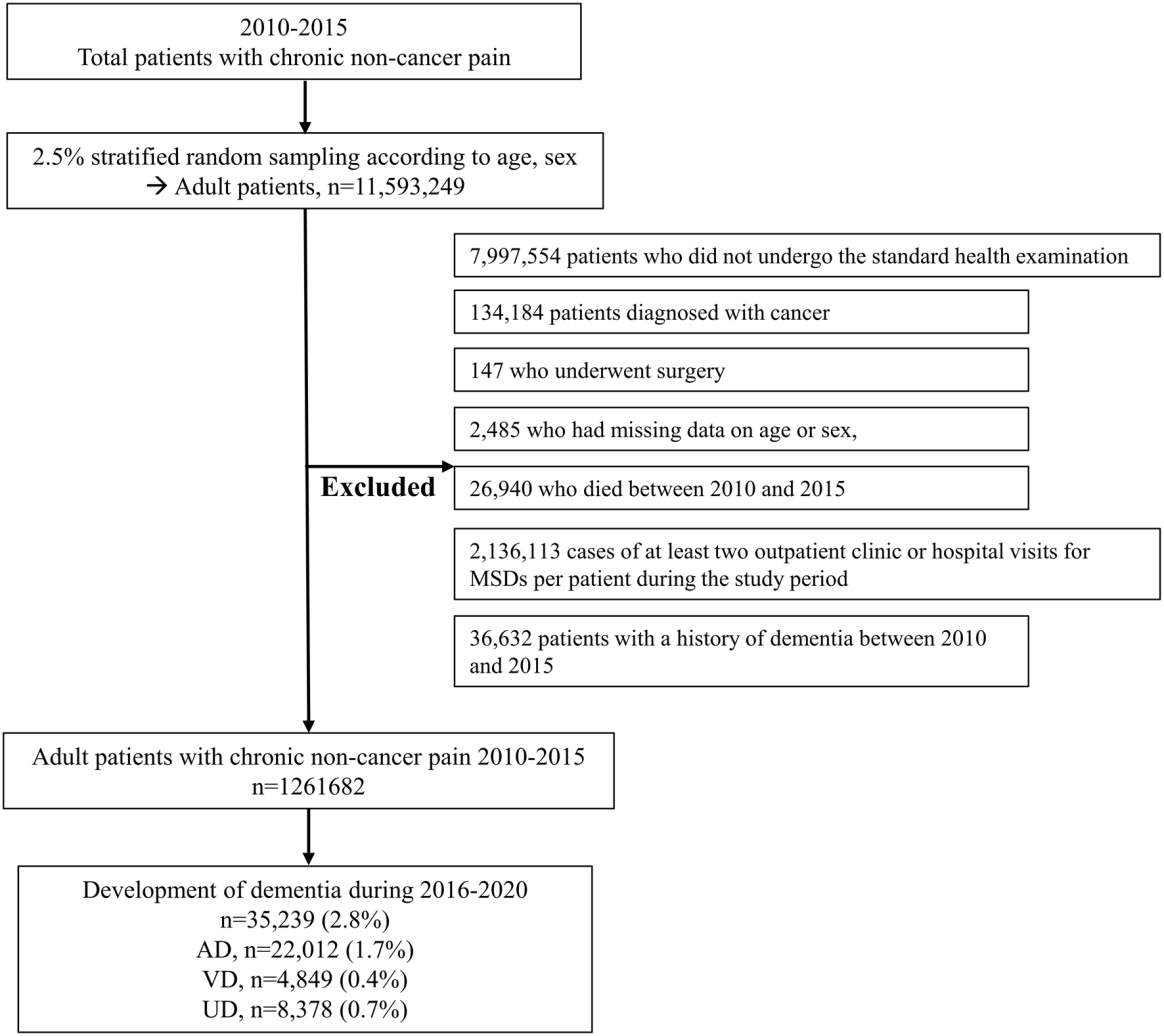
Flow chart of the selection process for patients with CNCP. *CNCP* chronic non-cancer pain; *MSD* musculoskeletal disease; *AD* Alzheimer's disease; *VD* vascular dementia; *UD* unspecified dementia.

Among these patients, 7,997,554 patients who did not undergo the standard health examination between 2010 and 2015 were excluded owing to the absence of data on important risk factors for stroke, such as body mass index (BMI), smoking status, or alcohol consumption^21^. Following this, 134,184 patients diagnosed with cancer, 147 who underwent surgery, 2,485 who had missing data on age or sex, and 26,940 who died between 2010 and 2015 were excluded. Furthermore, 2,136,113 cases of at least two outpatient clinic or hospital visits for MSDs per patient during the study period were excluded to focus exclusively on data on the final visit of each patient on the latest date because this study analyzed the development of dementia as a time-to-event analysis from January 1, 2016. In addition, 36,632 patients with a history of dementia between 2010 and 2015 were excluded. Therefore, 1,261,682 patients with CNCP were included in the final analysis (Fig. 1).

### Opioid use among patients with CNCP

In a systemic review of observational studies on the impact of long-term opioid therapy^22^, the minimum treatment duration of long-term opioid therapy was 90 days. Thus, among patients with CNCP, those who were prescribed opioids for ≥ 90 days between 2010 and 2015 were defined as opioid users in this study, whereas the others were considered as controls. All opioid prescriptions from outpatient clinics, long-term facility care centers, and acute-setting hospitals were captured in this study. Additionally, ≥ 90 days of opioid use was measured using the number of prescriptions filled in a specific time period. Specifically, to examine opioid prescriptions (≥ 90 days) between 2010 and 2015, the opioid prescription data from October 2009 to March 2016 was used to prevent people who actually received prescriptions of ≥ 90 days from being classified as controls. Opioid dosages were not provided in the dataset. Fentanyl, morphine, oxycodone, hydromorphone, methadone, codeine, dihydrocodeine, and tramadol were used to extract opioid prescriptions in this study. If any patients in the opioid group ceased using opioids until December 31, 2015, they were assigned to the control group.

### Study endpoint

The primary endpoint of this study was the development of dementia, evaluated from January 1, 2016, to December 31, 2020. According to the ICD-10 codes, dementia included AD (F00, G30), VD (F01), and unspecified dementia (UD) (F03); however, dementia due to other causative diseases (F02) was excluded. In South Korea, the data of all patients with dementia, regardless of its type, are registered in the NHIS database by physicians so that they may receive financial support for treatment expenses. There should therefore be no missing dementia registrations if all were treated for the disease.

### Measured confounders

Several confounders were identified in this study: Age and sex information was collected from the patient demographics. The BMI of patients was extracted and categorized into five groups: < 18.5, 18.5–24.9, 25.0–29.9, 30.0–34.9, and > 35.0 kg/m^2^. The smoking status of the patients was retrieved and divided into three categories: never smokers, previous smokers, and current smokers. Alcohol consumption status was categorized into three groups: no alcohol, mild alcohol, and heavy alcohol consumption, according to previous studies^23,24^. The mild-consumer group comprised patients with alcohol consumption ≤ 210 and ≤ 140 g/week in men and women, respectively, whereas the heavy-consumer group included patients with alcohol consumption > 210 and > 140 g/week in men and women, respectively. As part of the national health planning, South Koreans aged over 40 years are advised to have standardized health checkups every two years^25^. Information on BMI, history of smoking, and history of alcohol consumption was extracted using the 2014–2015 health checkup data for the patients in this study.

For socioeconomic status (SES)-related information, data regarding three factors (employment status, household income level, and residence) were gathered. The NHIS contains data on patients’ household income levels to determine their insurance premiums, and approximately 67% of the medical expenses are subsidized by the government^26^. However, individuals who have trouble paying their insurance premiums due to considerably low household incomes are enrolled in the Medical Aid Program, which covers almost all medical expenses to minimize the financial burden posed by treatment. Patients were divided into five groups based on quartile ratios: Q1–Q4 and the Medical Aid Program. Residences were classified as urban (Seoul and other metropolitan cities) or rural (all other areas). To adjust for patient comorbidity status, the Charlson comorbidity index was calculated using ICD-10 codes registered in 2010–2015 (Supplemental Digital Content 2). Moreover, data on patient disability status from 2010–2015 were extracted. All individuals with disabilities were registered in the NHIS database to enable them to receive benefits from South Korea’s social welfare system. Patients were initially categorized into six groups based on disability severity, and these groups were subsequently consolidated into two: (1) severe disability (patients with grade 1–3 disabilities) and (2) mild-to-moderate disability (those with grade 4–6 disabilities). Data on the regular use of other analgesics, such as pregabalin or gabapentin, paracetamol, and non-steroidal anti-inflammatory drugs, were collected as covariates.

### Statistical analysis

The clinicopathological characteristics of opioid users and the control group were compared using the t-test for continuous variables and the chi-squared test for categorical variables, respectively. We performed a univariable Cox regression analysis to examine whether opioid use was associated with the development of general dementia. In this time-to-event analysis, the diagnosis of dementia during 2016–2020 was considered an event, while the duration from January 1, 2016, to the first date of diagnosis of dementia until December 31, 2020, was considered time. Next, we fitted a multivariable Cox regression model to examine whether opioid use was independently associated with the development of general dementia. All covariates were included in the model for multivariable adjustment, and there was no multicollinearity issue between variables with the criterion of variance inflation factors < 2.0. Next, we divided general dementia into three types (AD, VD, and UD) and constructed three multivariable Cox regression models to investigate whether opioid use was independently associated with the development of the three types (AD, VD, and UD). Additionally, as a sensitivity analysis, multivariable Cox regression analyses for the development of dementia in patients with CNCP during 2017–2020 (except 2016), considering the latency between opioid use and dementia onset. Lastly, we performed subgroup analyses according to sex and age (< 60 years or ≥ 60 years) to examine whether the obtained results differed according to sex and age. The results of Cox regression analyses were presented as hazard ratios (HR) with 95% confidence intervals (CI), and log–log plots were used to confirm that the central assumptions of the Cox proportional hazard models were satisfied. All statistical analyses were performed using R software (version 4.0.3, R packages, R Project for Statistical Computing, Vienna, Austria). Statistical significance was set at *P* < 0.05.

## Results

### Study population

Table 1 shows the clinicopathological characteristics of the 1,261,682 patients with CNCP. The mean age was 50.0 years (standard deviation (SD):14.2 years), and the proportion of men was 49.8% (628,708/1,261,681). The number of opioid users among patients with CNCP was 21,800 (1.7%). From January 1, 2016, to December 31, 2020, 35,239 (2.8%) patients with CNCP were newly diagnosed with dementia. The proportions of AD, VD, and UD were 1.7% (22,012/1,261,682), 0.4% (4,849/1,261,682), and 0.7% (8,378/1,261,682), respectively, as shown in Fig. 1.

**Table 1:** Clinicopathological characteristics of the 1,261,682 patients with CNCP.

Variable	Mean value (SD) or number (%)
Age, year	50.0 (14.2)
Sex, men	628,708 (49.8)
BMI, kg/m^2^	
< 18.5	50,463 (4.0)
18.5–24.9	773,885 (61.3)
25.0–25.9	376,873 (29.9)
30.0–34.9	53,502 (4.2)
> 35.0	6,959 (0.6)
Smoking status	
Never smoker	782,757 (62.0)
Previous smoker	194,787 (15.4)
Current smoker	284,138 (22.5)
Alcohol consumption	
No alcohol consumption group	657,680 (52.1)
Mild alcohol consumption group	534,966 (42.4)
Heavy alcohol consumption group	69,036 (5.5)
Having a job	919,550 (72.9)
Household income	
Medical aid program	16,611 (1.3)
Q1 (lowest)	230,736 (18.3)
Q2	275,771 (21.9)
Q3	324,888 (25.8)
Q4 (highest)	384,174 (30.4)
Unknown	29,502 (2.3)
Residence	
Urban area	568,700 (45.1)
Rural area	692,982 (54.9)
Disability	
Mild to moderate	48,864 (3.9)
Severe	19,107 (1.5)
CCI, point	2.3 (2.0)
Opioid use	21,800 (1.7)
Other analgesic use	1,261,682
Gabapentin or pregabalin use	11,320 (0.9)
NSAIDs use	1,582 (0.1)
Paracetamol use	23,175 (1.8)
Underlying MSDs	
RA	34,411 (2.7)
OA	286,888 (22.7)
LBP	402,557 (31.9)
Neck pain	134,482 (10.7)
Gout	30,822 (2.4)
Other MSD	406,394 (32.2)

*CNCP* chronic non-cancer pain; *SD* standard deviation; *BMI* body mass index; *CCI* Charlson comorbidity index; *MSD* musculoskeletal disease; *NSAIDs* nonsteroidal anti-inflammatory drugs; *RA* rheumatoid arthritis; *OA* osteoarthritis; *LBP* low back pain.

### Comparison between opioid users and control group

Table 2 shows the results of the comparison of the clinicopathological characteristics between opioid users and the control group with CNCP. The prevalence of dementia was higher in opioid users (11.0%, 2,408/21,800) than in the control group (2.6%, 32,831/1,239,882). Specifically, the prevalence of AD (7.2%, 1,580/21,800), VD (1.3%, 278/21,800), and UD (2.5%, 550/21,800) in opioid users was higher than that of AD (1.6%, 2,0432/1,239,882), VD (0.4%, 4,571/1,239,882), and UD (0.6%, 7,828/1,239,882) in the control group.

**Table 2:** Comparison of the clinicopathological characteristics between opioid users and opioid-naïve patients with CNCP.

Variable	Opioid user n = 21,800	Control group n = 1,239,882	*P*-value
Age, year	63.8 (11.6)	49.7 (14.2)	< 0.001
Sex, male	8,266 (37.9)	620,442 (50.0)	< 0.001
BMI, kg/m^2^			< 0.001
< 18.5	606 (2.8)	49,857 (4.0)	
18.5–24.9	11,825 (54.2)	762,060 (61.5)	
25.0–25.9	7,901 (36.2)	368,972 (29.8)	
30.0–34.9	1,304 (6.0)	52,198 (4.2)	
> 35.0	164 (0.8)	6,795 (0.5)	
Smoking status			< 0.001
Never smoker	15,478 (71.0)	767,279 (61.9)	
Previous smoker	3117 (14.3)	191,670 (15.5)	
Current smoker	3205 (14.7)	280,933 (22.7)	
Alcohol consumption			< 0.001
No alcohol consumption group	16,269 (74.6)	641,411 (51.7)	
Mild alcohol consumption group	4813 (22.1)	530,153 (42.8)	
Heavy alcohol consumption group	718 (3.3)	68,318 (5.5)	
Having a Job	13,685 (62.8)	905,865 (73.1)	< 0.001
Household income			< 0.001
Medical aid program	1045 (4.8)	15,566 (1.3)	
Q1 (lowest)	4100 (18.8)	226,636 (18.3)	
Q2	3846 (17.6)	271,925 (21.9)	
Q3	5151 (23.6)	319,737 (25.8)	
Q4 (highest)	7218 (33.1)	376,956 (30.4)	
Unknown	440 (2.0)	29,062 (2.3)	
Residence			
Urban area	8838 (40.5)	559,862 (45.2)	< 0.001
Rural area	12,962 (59.5)	680,020 (54.8)	
Disability			
Mild to moderate	2980 (13.7)	45,884 (3.7)	< 0.001
Severe	819 (3.8)	18,288 (1.5)	
CCI, point	4.6 (2.4)	2.3 (2.0)	< 0.001
Other analgesic use			
Gabapentin or pregabalin use	2616 (12.0)	8,704 (0.7)	< 0.001
NSAIDs use	327 (1.5)	1,255 (0.1)	< 0.001
Paracetamol use	15,914 (73.0)	7,261 (0.6)	< 0.001
Total dementia	2408 (11.0)	32,831 (2.6)	< 0.001
AD	1580 (7.2)	20,432 (1.6)	< 0.001
VD	278 (1.3)	4,571 (0.4)	< 0.001
UD	550 (2.5)	7,828 (0.6)	< 0.001

*CNCP* chronic non-cancer pain; *BMI* body mass index; *CCI* Charlson comorbidity index; *MSD* musculoskeletal disease; *NSAIDs* nonsteroidal anti-inflammatory drugs; *RA* rheumatoid arthritis; *OA* osteoarthritis; *LBP* low back pain; *AD* Alzheimer's disease; *VD* vascular dementia; *UD* unspecified dementia.

### Multivariable Cox regression modeling

Table 3 shows the uni- and multivariable Cox regression analyses for the development of dementia in patients with CNCP. In multivariable model 1, opioid users showed a 15% (HR: 1.15, 95% CI: 1.10, 1.20; *P* < 0.001) higher risk of dementia than the control group. The opioid users showed a 16% (HR: 1.16, 95% CI: 1.10, 1.22; *P* < 0.001; model 2) and 15% (HR: 1.15, 95% CI: 1.06, 1.27; *P* = 0.001; model 4) higher risk of AD and UD, respectively, than the control group, while there was no statistical difference for VD (*P* = 0.359) between groups. The HRs of the 95% CIs of the other covariates in multivariable model 1 are presented in Supplemental Digital Content 3. Among covariates, < 18.5 kg/m^2^ (vs. 18.5–24.9 kg/m^2^) in BMI (HR: 1.17, 95% CI: 1.11, 1.23; *P* < 0.001) and current smoker (vs. never smoker) (HR: 1.17, 95% CI: 1.13, 1.22; *P* < 0.001) were associated with increased risk of total dementia, while alcohol consumption status was not (*P* > 0.05). The results of multivariable Cox regression analyses for the development of dementia in patients with CNCP during 2017–2020 (except 2016) were presented in Supplemental Digital Content 4, and similar results were obtained with the main results.

**Table 3:** Uni- and multivariable Cox regression analyses for the development of dementia in patients with CNCP.

Variable	HR (95% CI)	*P*-value
Unadjusted		
Total dementia		
Opioid user (vs control group)	4.36 (4.19, 4.55)	< 0.001
AD		
Opioid user (vs control group)	4.61 (4.38, 4.85)	< 0.001
VD		
Opioid user (vs control group)	3.61 (3.20, 4.07)	< 0.001
UD		
Opioid user (vs control group)	4.18 (3.83, 4.55)	< 0.001
Covariate-adjusted		
Total dementia (model 1)		
Opioid user (vs control group)	1.15 (1.10, 1.20)	< 0.001
AD (model 2)		
Opioid user (vs control group)	1.16 (1.10, 1.22)	< 0.001
VD (model 3)		
Opioid user (vs control group)	1.06 (0.94, 1.20)	0.359
UD (model 4)		
Opioid user (vs control group)	1.15 (1.06, 1.27)	0.001

*CNCP* chronic non-cancer pain; *HR* hazard ratio; *CI* confidence interval; *AD* Alzheimer's disease; *VD* vascular dementia; *UD* unspecified dementia.

### Subgroup analyses

Table 4 shows the results of subgroup analyses according to sex and age. Similar results were observed in both the male and female groups. However, opioid users in the ≥ 60 years old group showed a 20% (HR: 1.20, 95% CI: 1.12, 1.31; *P* < 0.001) higher risk of total dementia, while opioid users in the < 60 years old group did not show a significant difference compared with the control group (*P* = 0.060).

**Table 4:** Subgroup analyses according to sex and age.

Variable	HR (95% CI)	*P*-value
Male sex		
Total dementia		
Opioid user (vs control group)	1.13 (1.09, 1.18)	< 0.001
AD		
Opioid user (vs control group)	1.16 (1.09, 1.21)	< 0.001
VD		
Opioid user (vs control group)	1.03 (0.90, 1.18)	0.245
UD		
Opioid user (vs control group)	1.09 (1.04, 1.20)	0.002
Female sex		
Total dementia		
Opioid user (vs control group)	1.10 (1.06, 1.15)	< 0.001
AD		
Opioid user (vs control group)	1.13 (1.09, 1.17)	< 0.001
VD		
Opioid user (vs control group)	1.03 (0.90, 1.15)	0.524
UD		
Opioid user (vs control group)	1.07 (1.02, 1.12)	0.021
Age < 60 years old		
Total dementia		
Opioid user (vs control group)	1.05 (1.00, 1.09)	0.060
AD		
Opioid user (vs control group)	1.03 (0.98, 1.06)	0.143
VD		
Opioid user (vs control group)	0.98 (0.95, 1.03)	0.652
UD		
Opioid user (vs control group)	1.03 (0.99, 1.05)	0.205
Age ≥ 60 years old		
Total dementia		
Opioid user (vs control group)	1.20 (1.12, 1.31)	< 0.001
AD		
Opioid user (vs control group)	1.18 (1.15, 1.21)	< 0.001
VD		
Opioid user (vs control group)	1.05 (0.96, 1.14)	0.301
UD		
Opioid user (vs control group)	1.10 (1.05, 1.14)	< 0.001

*CNCP* chronic non-cancer pain; *HR* hazard ratio; *CI* confidence interval; *AD* Alzheimer's disease; *VD* vascular dementia; *UD* unspecified dementia.

## Discussion

In this population-based cohort study, we showed that among adult patients with CNCP, opioid users had a higher risk of developing dementia than the control group. This association was significant for AD but not VD in this study. Our results suggest that among opioid users with CNCP, prevention and management of dementia are important.

A few factors might have caused the elevated dementia risk in opioid users with CNCP. Persistent pain is a known risk factor for accelerated memory decline and dementia because pain directly compromises cognitive function^27^. The mechanisms of cognitive decline associated with chronic pain include decreased attentional capacity and impaired memory function^28^. Moreover, the stressful conditions caused by severe chronic pain might be related to faster cognitive decline via putative cortisol-based pathways^29^. Among the total number of patients with CNCP, opioid users might suffer from more severe and persistent pain than the control group, which would increase the risk of developing dementia.

Opioid usage is also related to CNS depression, which might be implicated in cognitive dysfunction in patients with CNCP^30^. CNS depression is related to neuroinflammation, which may play a central role in the pathophysiology of dementia^31^. Dublin et al. reported that people with the heaviest opioid use had a slightly higher dementia risk than those with little or no use in community settings^14^. Donovan et al. recently reported that 29.8% of 737,839 community-dwelling beneficiaries with dementia in the United States were prescribed opioid analgesics^32^. Christina et al. reported that opioid use in the elderly Danish population was frequent, particularly in patients with dementia^33^. In addition to these previous studies^14,32^, our results showed that opioid users were at a higher risk for the development of dementia, especially AD, among patients with CNCP in South Korea.

In subgroup analyses, opioid use was associated with an increased risk in the old age group (≥ 60 years). Elderly people are vulnerable to dementia risk, and it is a significant public health problem nowadays^34^. A recent national cohort study in Israel reported that people of ages 75 to 80 who were exposed to opioids had a higher risk of dementia among elderly people aged 60 years and over^16^, and it suggested that people of older age who were prescribed opioids could be a high-risk population for the development of dementia. Our results also suggested that policymakers, physicians, or caregivers should consider dementia risk according to opioid exposure.

The significant association between opioid use and AD, but not VD, is an important finding of this study. In a recent cohort study in the United States, inappropriate opioid prescriptions were common in patients with AD^35^. The pathophysiology of VD is related to the anatomical location of tissue changes as well as the time after the initial vascular event, such as an infarction of the brain^36^. The risk factors for VD were old age, diabetes mellitus, hypertension, atherosclerosis, and a history of stroke^37^. Therefore, vascular events might be more important for the development of VD than opioid usage or pain intensity among patients with CNCP. Although long-term prescriptions of opioids might be linked to an increased risk of stroke^38^, which is related to an increased risk of VD^37^, the direct relationship between opioid use and the risk of VD has not been identified. Previous literature focused on the relationship between opioid use and the risk of total dementia or AD^14–17^. We divided total dementia into AD, VD, and UD and found that VD was not associated with opioid use. However, the information regarding this issue is still lacking, and more studies are needed.

Among the covariates in Supplemental Digital Content 3, there are some important findings. Current smoking status, unemployment, and low household income (Medical Aid Program) were independent risk factors for dementia development. Current smoking is a well-known risk factor for the development of AD and other types of dementia, such as VD, among older adults^39^. In a recent cohort study in Denmark, low SES was identified as a risk factor for the development of dementia^40^. As we reported, SES inequity is an important issue for the early detection and treatment of dementia. Moreover, < 18.5 kg/m^2^ in BMI (HR: 1.17, 95% CI: 1.11) and current smoker were associated with an increased risk of total dementia. Malnutrition was associated with an increased risk of cognitive decline^41^, which is related to an increased risk of dementia. Smoking was also a well-known risk factor for the development of dementia^42^, as we found in this study.

This study had several limitations. First, we could not evaluate and adjust for the severity of MSD. For example, the duration and severity of pain in each MSD may influence the use of opioids and lead to an increased prevalence of dementia among patients with CNCP. Second, as we used opioid prescription data to determine its users, the actual compliance of opioid use among users was not evaluated in this study. Third, although we adjusted for many covariates in the multivariable Cox regression modeling, there might be some residual and unmeasured confounders that could affect the results. Fourth, we did not consider the daily dosage and duration of opioid prescriptions in the opioid user group, which might affect the results. Fifth, there might be a distribution bias because only 21,800 (1.7%) were opioid users among a total of 1,261,682 patients with CNCP. However, the results of this study are valuable because we used a large sample size and followed up over a long period to detect the development of dementia using time-to-event analysis.

In conclusion, among adult patients with CNCP in South Korea, opioid users were at a higher risk of developing dementia than the control group. Moreover, this association was significant for AD but not for VD in this study. Our results suggest that in patients with CNCP, public health strategies should target opioid users for early dementia detection and intervention.

### Supplementary Information

Supplementary Information 1.

Supplementary Information 2.

Supplementary Information 3.

Supplementary Information 4.

## Data Availability

Data will be available upon reasonable request to corresponding author.
