# Supplementary material for: Impact of prescribed opioid use on development of dementia among patients with chronic non-cancer pain
Source: Sci Rep. 2024 Feb 9;14:3313. doi: 10.1038/s41598-024-53728-3 (PMC10853162; doi:10.1038/s41598-024-53728-3)
Supplement: Supplementary file 3 — Supplementary Information 3. [file 41598_2024_53728_MOESM3_ESM.docx]

Supplemental Digital Content 3. HRs of the 95% CIs of the other covariates in multivariable model 1

| Variable | | HR (95% CI) | *P*-value |
| --- | --- | --- | --- |
| Age, year | | 1.12 (1.12, 1.12) | <0.001 |
| Sex, male | | 0.86 (0.84, 0.89) | <0.001 |
| BMI, kg/m^2^ | |  |  |
|  | <18.5 | 1.17 (1.11, 1.23) | <0.001 |
|  | 18.5-24.9 | 1 |  |
|  | 25.0-25.9 | 0.95 (0.93, 0.97) | <0.001 |
|  | 30.0-34.9 | 0.93 (0.88, 0.98) | 0.009 |
|  | >35.0 | 1.01 (0.86, 1.19) | 0.910 |
| Having a Job | | 0.96 (0.94, 0.98) | <0.001 |
| Smoking status | |  |  |
|  | Never smoker | 1 |  |
|  | Previous smoker | 0.98 (0.94, 1.01) | 0.213 |
|  | Current smoker | 1.17 (1.13, 1.22) | <0.001 |
| Alcohol consumption | |  |  |
|  | No alcohol consumption group | 1 |  |
|  | Mild alcohol consumption group | 0.97 (0.84, 1.15) | 0.852 |
|  | Heavy alcohol consumption group | 1.03 (0.97, 1.10) | 0.336 |
| Household income | |  |  |
|  | Medical aid program | 2.37 (2.21, 2.53) | <0.001 |
|  | Q1 (lowest) | 1 |  |
|  | Q2 | 1.01 (0.97, 1.04) | 0.746 |
|  | Q3 | 0.97 (0.94, 1.01) | 0.106 |
|  | Q4 (highest) | 0.97 (0.94, 0.99) | 0.030 |
|  | Unknown | 1.00 (0.93, 1.08) | 0.993 |
| Residence | |  |  |
|  | Urban area | 1 |  |
|  | Rural area | 1.10 (1.08, 1.13) | <0.001 |
| Disability | |  |  |
|  | Mild to moderate | 1.22 (1.18, 1.26) | <0.001 |
|  | Severe | 1.69 (1.61, 1.78) | <0.001 |
| CCI, point | | 1.13 (1.12, 1.13) | <0.001 |
| Pregabalin or gabapentin use | | 1.09 (1.03, 1.15) | 0.003 |
| Paracetamol use | | 1.08 (0.92, 1.26) | 0.344 |
| Non-steroidal anti-inflammatory drugs use | | 1.02 (0.89, 1.16) | 0.786 |
| Underlying MSDs | |  |  |
|  | RA | 0.97 (0.92, 1.01) | 0.161 |
|  | OA | 1.12 (1.10, 1.15) | <0.001 |
|  | LBP | 1.14 (1.11, 1.17) | <0.001 |
|  | Neck pain | 1.05 (1.02, 1.08) | 0.001 |
|  | Gout | 0.98 (0.93, 1.04) | 0.471 |
|  | Other MSD | 1.05 (1.02, 1.07) | <0.001 |
| Year | |  |  |
|  | 2010 | 1 |  |
|  | 2011 | 0.87 (0.82, 0.93) | <0.001 |
|  | 2012 | 0.76 (0.71, 0.80) | <0.001 |
|  | 2013 | 0.71 (0.67, 0.75) | <0.001 |
|  | 2014 | 0.59 (0.56, 0.62) | <0.001 |
|  | 2015 | 0.50 (0.48, 0.53) | <0.001 |

HR, hazard ratio; CI, confidence interval; BMI, body mass index; CCI, Charlson comorbidity index; MSD, musculoskeletal disease; RA, rheumatoid arthritis; OA, osteoarthritis; LBP, low back pain
