# Supplementary material for: Impact of prescribed opioid use on development of dementia among patients with chronic non-cancer pain
Source: Sci Rep. 2024 Feb 9;14:3313. doi: 10.1038/s41598-024-53728-3 (PMC10853162; doi:10.1038/s41598-024-53728-3)
Supplement: Supplementary file 4 — Supplementary Information 4. [file 41598_2024_53728_MOESM4_ESM.docx]

Supplemental Digital Content 4. Multivariable Cox regression analyses for the development of dementia in patients with CNCP during 2017-2020 considering latency time of opioid use

| Variable | | | HR (95% CI) | *P*-value |
| --- | --- | --- | --- | --- |
|  | Total dementia (model 1) | |  |  |
|  | | Opioid user (vs control group) | 1.12 (1.08, 1.17) | <0.001 |
|  | AD (model 2) | |  |  |
|  | | Opioid user (vs control group) | 1.15 (1.08, 1.19) | <0.001 |
|  | VD (model 3) | |  |  |
|  | | Opioid user (vs control group) | 1.04 (0.92, 1.17) | 0.472 |
|  | UD (model 4) | |  |  |
|  | | Opioid user (vs control group) | 1.10 (1.03, 1.19) | 0.005 |

CNCP, chronic non-cancer pain; HR, hazard ratio; CI, confidence interval; AD, Alzheimer's disease; VD, vascular dementia; UD, unspecified dementia
